# Evidence based anti-osteoporosis effects of *Periplaneta americana* L on osteoblasts, osteoclasts, vascular endothelial cells and bone marrow derived mesenchymal stem cells

**DOI:** 10.1186/s12906-017-1917-7

**Published:** 2017-08-18

**Authors:** Yan-Fen Huang, Long-Jian Li, Si-Qian Gao, Yang Chu, Jie Niu, Fu-Neng Geng, Yong-Mei Shen, Li-Hua Peng

**Affiliations:** 1 Institute of Pharmaceutics, College of Pharmaceutical Sciences, Zhejiang University, 866# Yuhangtang Road, Hangzhou City, 310058 People’s Republic of China; 2 State Key Laboratory of Quality Research in Chinese Medicine, Macau University of Science and Technology, Macau, People’s Republic of China; 3 Zhejiang Provincial Corps Hospital of Chinese People’s Armed Police Forces, Jiaxing, Zhejiang People’s Republic of China; 4 Sichuan Engineering Research Center for Medicinal Animals, Yingmenkou Road 88#, Chengdu City, Sicuan Province People’s Republic of China; 5 GOODDOCTOR Pharmaceutical Company, Yingmenkou Road 88#, Chengdu City, Sicuan Province People’s Republic of China

**Keywords:** *Periplaneta americana* L, Anti-osteoporosis, Osteoblasts, Osteoclasts, HUVECs, BMSCs

## Abstract

**Background:**

Kangfuxin (KFX) is the ethanol extract of *Periplaneta americana* L, which has been widely used in the Traditional Chinese Medicine for the repair and regeneration of injured organ and tissues with long history. This study is to investigate the influence of KFX in the various cellular activities and evaluate the anti-osteoporosis potential of KFX.

**Methods:**

The influence of the KFX in the cellular activities, including: 1) migration, osteocalcin secretion of osteoblasts; 2) apoptosis of osteoclasts; 3) migration and tube formation of human umbilical vein endothelial cell (HUVEC); and 4) proliferation, cell cycle regulation and migration of bone marrow mesenchymal stem cells (BMSCs), were investigated systematically.

**Results:**

KFX was shown to significantly 1) Promote of the migration of osteoblasts, HUVEC, and BMSCs; 2) Increase the secretion of osteocalcin and mineralization of osteoblasts; 3) Accelerate the apoptosis of osteoclasts; 4) Stimulate the proliferation and regulate the cell cycle of BMSCs.

**Conclusion:**

Taken together, these results provide the evidence for the osteogenesis, anti-osteoporosis and angiogenesis effects of KFX, with the mechanism of activating the bone formation through stimulating the osteoblasts and HUVECs, as well as inhibiting the bone absorption by inhibiting the osteoclasts activities. The KFX was definitely shown a promising bone turnover agent with great potential for anti-osteoporosis treatment.

## Background

Osteoporosis, the most frequent bone remodeling disease, is characterized by a low bone mass with susceptibility to fracture, which results in an increased risk of adverse health outcomes including decreased quality of life, disability, recurrent hospitalizations, and death [1, 2]. In recent years, this disease has become a major health hazard afflicting over 2000 million people worldwide. Therefore, prevention and early treatment of osteoporosis are important to avoid its complications, especially for those osteopenic postmenopausal women.

There are some anti-osteoporotic drugs that could prevent and treat osteoporosis. However, many of these drugs are associated with serious side effects. For several years, estrogen replacement therapy has been used to prevent osteoporosis in postmenopausal women [3], but studies have also reported that estrogen replacement therapy were associated with headache and increased cancer risk such as breast cancer, endometrial cancer, and ovarian cancer [4, 5]. The other example is alendronate, which is associated with osteonecrosis of jaws, increase in the risks of atypical odd fractures and incidence of oesophageal cancer after long-term consumption [6]. Current therapeutics in clinical use included bisphosphonates, anti-RANKL, parathyroid hormone, and strontium ranelate, which may reduce the risk of first and recurrent fractures [7]. Therefore, there is an urgent and increasing need for effective pharmacotherapy.

Kangfuxin (KFX), the ethanol extract of *Periplaneta americana* L (Dictyoptera; Blattidae) has been exploited as Traditional Chinese Medicine (TCM) for over hundreds of years against disorders such as hepatitis, trauma, gastric ulcers, burns, and heart diseases. Shen Nong’s Herbal Classic has recorded that *Periplaneta americana* L is a traditional medicinal insect possesses many therapeutic activities, for examples, promoting blood circulation, nourishing yin and myogenic effects. It is widely used in clinic in China to treat various diseases, such as blood stasis of bedsore, peptic ulcer disease, trauma, burns by improving local blood circulation, eliminating edema, reducing the local inflammatory reaction, alleviating exudation and promoting wound healing. Given the aforementioned findings, KFX may have therapeutic effects in bone diseases, for example, osteoporosis. However, the influence of KFX in osteoporosis has not been reported elsewhere. In the bone environment, bone metabolism is a dynamic process of bone remodeling that is dependent on the balance between osteoblast-driven bone formation and osteoclast- mediated bone resorption [8]. Meanwhile, angiogenesis, also known as neovascularization, is a process that forms new blood vessels from existing vessels, which is of physiological and pathological importance [9]. To regenerate the osteoporotic defects and the bone defects, the angiogenesis and neovascularization are critical processes which provide nutrients and renewable autologous cells to heal the defect. It is well known that VEGF is an angiogenic growth factor which regulates all the critical steps of angiogenic process [10–13]. Recent studies also shown that BMSCs played important roles in regulating the bone regeneration process, in which the BMSCs could be mobilized and they exert reparative effects at the site of bone defect [14, 15]. Therefore, in the present study, with a commercially available product, Xianlingubao, prepared from Chinese herbal formula extract that has been widely used for the osteoporosis treatment in clinic in China as a positive control (PC), the anti-osteoporosis potential of the KFX in the four important cell models, including osteoblast, osteoclast, HUVEC and BMSCs were investigated.

## Methods

### Materials

KFX is a commercially available product supplied by the collaborator, Haoyisheng Pharmaceutical Company (Chengdu, Sichuan Province, China), who is the manufacturer of KFX product. Xianlingubao is bought from the Huqinyutang Drugstore. Dulbecco Modified Eagle’s Media DMEM (Gibco, USA), fetal bovine serum (Gibco, USA). MTT reagents [(3-(4,5-dimethylthiazol-2-yl)-2,5-diphenyltetrazolium bromide and ECM gel were purchased from Sigma-Aldrich, USA. Cell Cycle, Apoptosis Analysis Kit and Alkaline Phosphatase Diethanolamine Activity Kit were purchased from Beyotime. Osteocalcin ELISA Assay Kit (EAGLE Bioscience, OST31-K01), Alizarin red’s kit were purchased from Genmed. Transwell plates (Corning #342) were purchased from FC500MCL, Bechman coult. Osteoblast-like UMR-106 cells, murine mono- cyte/macrophage RAW264.7 cells, and human umbilical vein endothelial cells (HUVEC) were acquired from the American type Culture Collection (ATCC, Manassas, VA).

### Preparation of KFX

*Periplaneta americana* L was obtained from the Good Agriculture Practice (GAP) breeding base, Sichuan, China. The dried and powered *Periplaneta americana* L (200 g) was extracted with 90% ethanol (1.2 L) twice at 80∘C. After filtration through 0.22 μm filter membranes and concentrated under reduced pressure and lyophilized into power at a yield of 10% by weight of the starting materials, it was stored at −20∘C until use. Major active compounds of KFX, uracil and hypoxanthine, were detected and quantified by Ultra Performance Liquid Chromatography (UPLC) with external standards. The quantity of Uracil and hypoxanthine in the extract was 0.2 mg/ml and 0.6 mg/ml, respectively. The whole study was performed with the product of the same batch.

### Osteoblast migration assay

Osteoblast migration assays were performed using Culture-Insert u-Dishes as described by the manufacturer (Ibidi).70ul suspension solution of Osteoblasts (5x10^5^cells/ml) were seeded into the inner well of the u-Dish and incubated at 37 °C and 5% CO_2_ overnight. The supernatant and migration chamber inserts were removed, unattached cells were rinsed off, then osteoblasts were incubated with 2 ml serum-free DMEM medium containing KFX (0.1, 1, 2 mg/ml) and PC substance (0.1 and 1 mg/ml), and the blank control group treated with 2 ml serum-free DMEM medium. Osteoblast migration distance was determined by microscopic imaging at 0, 24 and 48 h after post treatment. The wound healing percentage was analyzed using Image J analysis software and calculated with the following formula:

$$ \mathrm{Wound}\  \mathrm{healing}\%=100\%-\left(\mathrm{Remaining}\  \mathrm{cell}\  \mathrm{free}\  \mathrm{area}/\mathrm{Initial}\  \mathrm{cell}\  \mathrm{free}\  \mathrm{area}\right)\% $$

### Mineralized nodule formation in osteoblast

Levels of mineralized nodule formation were evaluated as previously described [16]. Briefly, osteoblasts were seeded at a density of 5× 10^4^ cells/wells in 24-well culture plated and incubated at 37 °C with 5% CO_2_ overnight to form a confluent monolayer. The grown medium was replaced with medium containing various concentrations of KFX (0.1, 1 and 2 mg/ml) and PC substance ((0.1, 1 and 2 mg/ml) and incubated for 7 days at 37 °C and 5% CO_2_. The group incubated with 1 ml medium was the blank control group. After 7 days, calcium deposition was determined using alizarin red-S staining. Briefly, cells were washed with PBS, followed by fixation in ice cold 70% ethanol for at least 1 h. Ethanol was removed and cells were rinsed with water and stained with 40 mM Alizarin-red S (pH 4.2) for 10 min at room temperature. Stained cells were further processed by five rinses with water, followed by a 15-min wash in PBS with rotation to reduce nonspecific Alizarin-red stain. Stained cultures were photographed under a microscope (Eclipse Ti-E/U/S; Nikon).

### BGP level in osteoblast

BGP was measured from cell culture medium using mouse osteocalcin ELISA Assay Kit (EAGLE Bioscience, OST31-K01) according to the manufacturer’s protocol. Briefly, osteoblasts were seeded at a density of 6.67 × 10^4^ cells/ml (3 ml per well) in 6 well culture plate and incubated at 37 °C with 5% CO_2_ overnight to form a confluent monolayer. Then, the grown medium was replaced with medium containing various concentrations of KFX (0.1, 1 and 2 mg/ml) and PC substance ((0.1, 1 and 2 mg/ml) and incubated for 72 h. At 72 h to take on the 0.5 ml cell supernatant and saved at −20 °C. Subsequently, osteocalcin standards and these samples were placed in 96-well ELISA microtiter plates (100ul per well) coated with monoclonal detective antibodies and incubated for 90 min at 37 °C. Then the osteocalcin antiserum were placed in 96 well plates (100ul per well) and incubated for 60 min at 37 °C, followed by three washes with TBS (0.01 M). After that, 100ul ABC reagent were placed in well plate and incubated for 30 min at 37 °C, followed by five washes with TBS (0.01 M). 100ul of TMB solution were added and incubated for 30 min at 37 °C and kept in dark place. After adding stop solution, absorbance was measured at 405 nm. Results are presented as the percentage of change of the activity compared with the untreated controls.

### Phosphatase (ALP) level in osteoblast

To determine the secretion of ALP stimulated by KFX, the osteoblast were seeded in 24-well plates at a density of 5 × 10^4^ cells/well and cultured in DMEM with various concentrations of KFX (0.1, 1 and 2 mg/ml) and PC substance ((0.1, 1 and 2 mg/ml). the levels of ALP activity was performed according to the manufacturer’s instruction (Beyotime, Jiangsu, China). While the levels of ALP activity at day 7 of cell culture were determined by measuring the transformation of p-nitrophenyl-phosphate into p-nitrophenol. Briefly, the cells were detached from the plates using trypsin/EDTA, and centrifuged for 5 min at 1000 rpm after being washed twice with PBS. The cells were resuspended in lysis buffer with 0.2% NP-40. ALP activity was determined by absorbance at 405 nm using pNPP as the substrate. All experiments were performed in triplicate to obtain the average data.

### Osteoclast apoptosis assay

An annexin V-FITC apoptosis detection Kit (Beyotime, C1063) was used for Osteoclast assessing apoptosis. In the process of induction from RAW264.7 to osteoclasts, RAW264.7 cells were pre-incubated in DMEM supplemented with 10% FBS and 1% penicillin/streptomycin. In order to induce RAW264.7 to osteoclasts, RAW264.7 cells (2 × 10^5^cells per well) were cultured in the presence of receptor activator of NF-kB ligand (RANK-L, 100 ng/ml) for 7 days. The culture medium was replaced on days 3 and 5. After 7 days, RAW264.7 cells were induced to osteoclasts. Subsequently, the medium were removed and cells were incubated with a dose for KFX (0.1, 1 and 2 mg/ml) with serum-free medium and PC substance (0.1, 0.5 and 1 mg/ml) with serum-free medium for 72 h at 37 °C and 5% CO_2_. The experiments were performed in triplicate and 3 ml per well. Then the cell apoptosis assay was performed using Annexin V-FITC Apoptosis Detection Kit following the manufacturer’ instruction. Osteoclasts were harvested using 0.25% trypsin, washed once in PBS, re-suspended and incubated with 200ul Annexin V-FITC and 10ul propidium iodide for 20 min at room temperature in the dark following the manufacturer’s instructions, which were analyzed with a flow cytometer (FC500MCL, Bechman coult). Data are expressed as the mean ± standard deviation (SD) from three independent experiments. All the data in each assay were determined by student’s t-t test to compare the difference of each tested group with the blank control group.

### HUVEC chemotaxis assay

Endothelial cells analyzed with the aid of a transwell. In a word, the endothelial cells suspension 1 × 10^4^ cells/well in 100ul, is added to the upper compartment of the 24 transwell plate, (Corning #3422). A dose response for KFX (0.1, 1 and 5 mg/ml) and PC substance (0.1, 1 and 5 mg/ml) are added to the lower chamber in 600ul medium, the group treated with medium as blank control. The transwell plate is then incubated for 24 h at 37 °C and 5% CO2. Hereafter, the cells remaining in the upper chamber were removed and cells on the under surface of the filters were fixed with 4% paraformaldehyde for 30 min. The migrated cells were stained with 5% crystal violet dye solution for 20 min, washed with PBS, and then photographed under a microscope (Eclipse Ti-E/U/S; Nikon).

### Tube formation of HUVEC

Tube formation assay was performed with some modifications according to the earlier literature [17]. Extracellular matrix (ECM) gel was diluted to 5 mg/mL with serum-free medium and mixed to homogeneity. Then, 50 μL diluted ECM gel was added to 96-well plate in each well and incubated at 37 °C for 30 min for hardening. At the same time, a dose for KFX (0.1, 1 and 2 mg/ml) with serum-free medium and PC substance (0.1, 1 and 2 mg/ml) with serum-free medium should first be performed. After that, endothelial cells were harvested with trypsin and re-suspended in 5 × 10^4^ cells per mL with previously performed concentrations of KFX and PC substance. 200 μL medium containing cells were gently added to the matrix-coated plates and incubated at 37 °C for 18 h. The capillary-like structures were then photographed at 18 h with a digital microscope.

### BMSC proliferation assay

The isolation and culture of primary BMSCs was performed according to the method previously described [18, 19]. Three-week-old Sprague-Dawley male rats (50–60 g) were supplied by Zhejiang University Experimental Animal Center, China. Briefly, rat femurs were cut away from the epiphysis, and bone marrow was flushed out by using a syringe filled with Dulbecco’s modified Eagle’s medium (DMEM; Gibco, USA) supplemented with 10% fetal bovine serum (FBS; Hyclone, USA), Lglutamine, penicillin (50 U/ml), and streptomycin (50 U/ml). The cell suspension was placed into 25-cm2 flasks (IWAKI Glass, Japan) and cultured at 37 °C under 5% CO2. The medium was changed on day 4 of culture and every 3 days thereafter. When the cells of the first passage became sub-confluent, the cells were detached from the flask by using treatment, for 5 min at 37 °C, with phosphate-buffered saline (PBS) containing 0.25% (wt) trypsin and 0.02% (wt) ethylene-diamine tetraacetic acid. Fourth passage cells at sub-confluence were used for all experiments.

Cell proliferation was determined by 3-[4,5-dimethylthiazol-2-yl]-2,5-diphenyltetrazoliumbromide (MTT) assay [20]. Briefly, BMSCs were seeded at a density of 2.5 × 10^4^ cells/wells in 96-well culture plate and incubated at 37 °C with 5% CO_2_ overnight to form a confluent monolayer. Then, the grown medium was replaced with medium containing various concentrations of KFX (0.01, 0.05, 0.25, 1.25, 6.25 and 31.25 mg/ml) and PC substance (0.01, 0.05, 0.25, 1.25, 6.25 and 31.25 mg/ml) and incubated for 24 h. Subsequently, 40 μl of a 5 mg/ml MTT solution in medium were added to 200ul of medium in culture wells and incubated for 4 h at 37 °C. Finally, the MTT containing media was removed and the insoluble purple formazan crystals produced by live cells were dissolved in 100 μL of dimethyl sulfoxide (DMSO, Sigma-Aldrich, Milwaukee, WI). The plate was placed on a rocking shaker for at least 10 min and then the purple DMSO solution in each well was monitored at 570 nm using an enzyme-linked immunometric meter reader. The experiments were performed in sextuplicate.

### BMSCs cell cycle analysis

BMSCs were seeded at a density of 1 × 10^5^cells/wells in DMEM complete media in six well tissue culture plate. The old media was changed by starved DMEM media (0.2% FBS) and kept for another 12 h. Starved BMSCs were incubated with: i) KFX (0.05, 1.25 and 6.25 mg/mL), ii) PC substance (0.05, 1.25 and 6.25 mg/mL) for 24 h. FACS analysis of treated and untreated cells was performed by PI staining in a flow cytometer according to the standard literature. Extent of propidium iodide staining of the gated population was displayed in a histogram and the regions are defined as: G0/G1, S, and G2/M. The data were analyzed using FCS Express V3 software [21].

### BMSCs migration assay

For the cell migration assay, BMSCs suspension 1 × 10^4^ cells/well in 100ul, is added to the upper compartment of the 24 transwell plate, (Corning #3422). A dose response for KFX (0.1, 1 and 5 mg/ml) and PC substance (0.1, 1 and 5 mg/ml) are added to the lower chamber in 600ul medium, the group treated with medium as blank control. The transwell plate is then incubated for 24 h at 37 °C and 5% CO_2_. Subsequently, the cells remaining in the upper chamber were removed and cells on the under surface of the filters were fixed with 4% paraformaldehyde for 30 min. The migrated cells were stained with 5% crystal violet dye solution for 20 min, washed with PBS, and then photographed under a microscope (Eclipse Ti-E/U/S; Nikon).

### Statistical analysis

Data are expressed as mean ± SD from at least 3 independent experiments. Statistical analyses were performed using the Students t-test to analyze differences among groups. *P* < 0.05 was considered statistically significant.

## Results

### Effect of KFX on osteoblasts

#### Osteoblasts migration assay

**Fig. 1:**
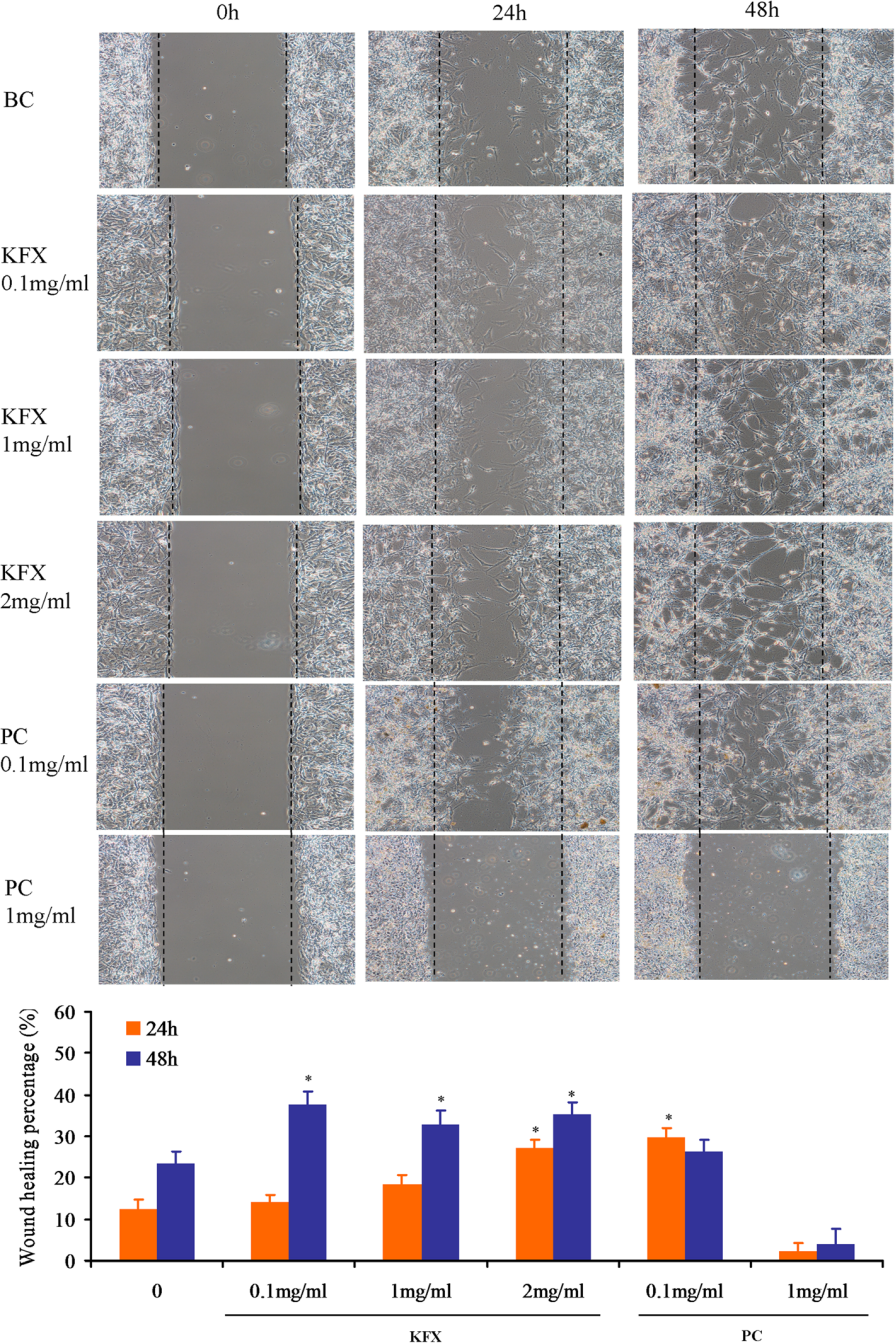
Osteoblasts migration assay. The migration of osteoblasts was measured with wound scratch assay. Osteoblasts were treated with KFX (0.1, 1 and 2 mg/mL), or PC substance (0.1 and 1 mg/mL). Significant migration was observed in cells treated with KFX (0.1, 1 and 2 mg/mL) compared with the blank control group. The quantitative evaluation and statistical analysis of wound closure percentage in wound scratch assay measured by Image J software. Results are expressed as means ± SD of three experiments (*P < 0.05, versus blank control)

The osteoblasts migration is one of the key steps of bone formation process. Therefore, the migration of osteoblasts was monitored in relation to the closure of a denuded area scratched in a confluent monolayer. From the Fig. 1, after treated with 24 h, both KFX (0.1, 1, 2 mg /mL) and PC substance (0.1 mg/mL) induced the obvious stimulation in the cells migration (*p* < 0.05), compared to the blank control. As time extended to 48 h, KFX (0.1, 1, 2 mg/mL) displayed a further enhancement in the osteoblasts migration (*p* < 0.05), consistent with that expressed by PC substance (0.1 mg/mL, *p* < 0.05). However, the high concentration of PC substance (1 mg/mL) did not show stimulation in cells migration. These results altogether demonstrate that KFX could markedly enhance the migration of osteoblasts within the 0.1-2 mg/mL concentration range, indicating its potential in facilitating the new bone formation.

#### Alizarin red S staining for mineralization

**Fig. 2:**
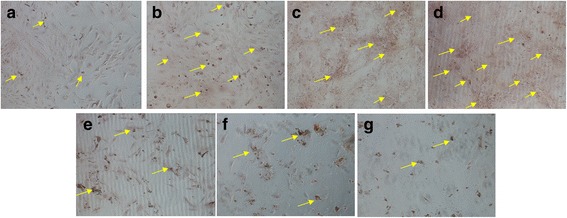
Levels of mineralized nodule formation assay. Osteoblasts were incubated with different concentrations of KFX and positive control for 7 days and then stained with Alizarin-S red as a measure of osteoblast mineralization. **a** Blank control (untreated), **b** KFX (0.1 mg/mL), **c** KFX (1 mg/mL), **d** KFX (2 mg/mL), **e** PC substance (0.1 mg/mL), **f** PC substance (1 mg/mL), **g** PC substance (2 mg/mL). Some calcium nodules were headed with yellow arrows

Calcification occurs at nucleation sites known as matrix vesicles present in the lacunae of mineralizing cartilage. It is important process to accumulate Ca^2+^ and inorganic phosphate which serve as nucleating agents for the formation of hydroxyapatite, the main inorganic component of bone. Calcification is widely used to indicate the recovery of bone healing. Therefore, Alizarin red S (ARS) staining, that has been used for decades to evaluate calcium-rich deposits by cells in culture was investigated in the tested osteoblasts. As it was shown in Fig. 2b–d, in the osteoblasts incubated with KFX (0.1, 1, 2 mg/mL), large area of positive staining, indicated the wide formation of calcium nodules were shown. By contrast, in the blank control and PC substance groups (0.1, 1, 2 mg/mL, Fig. 2e–g), much less calcium nodules were observed, which reminded the advantageous effect of KFX in promoting the calcification in osteoblasts for bone regeneration.

#### Osteocalcin (BGP) level and ALP activity assay

**Fig. 3:**
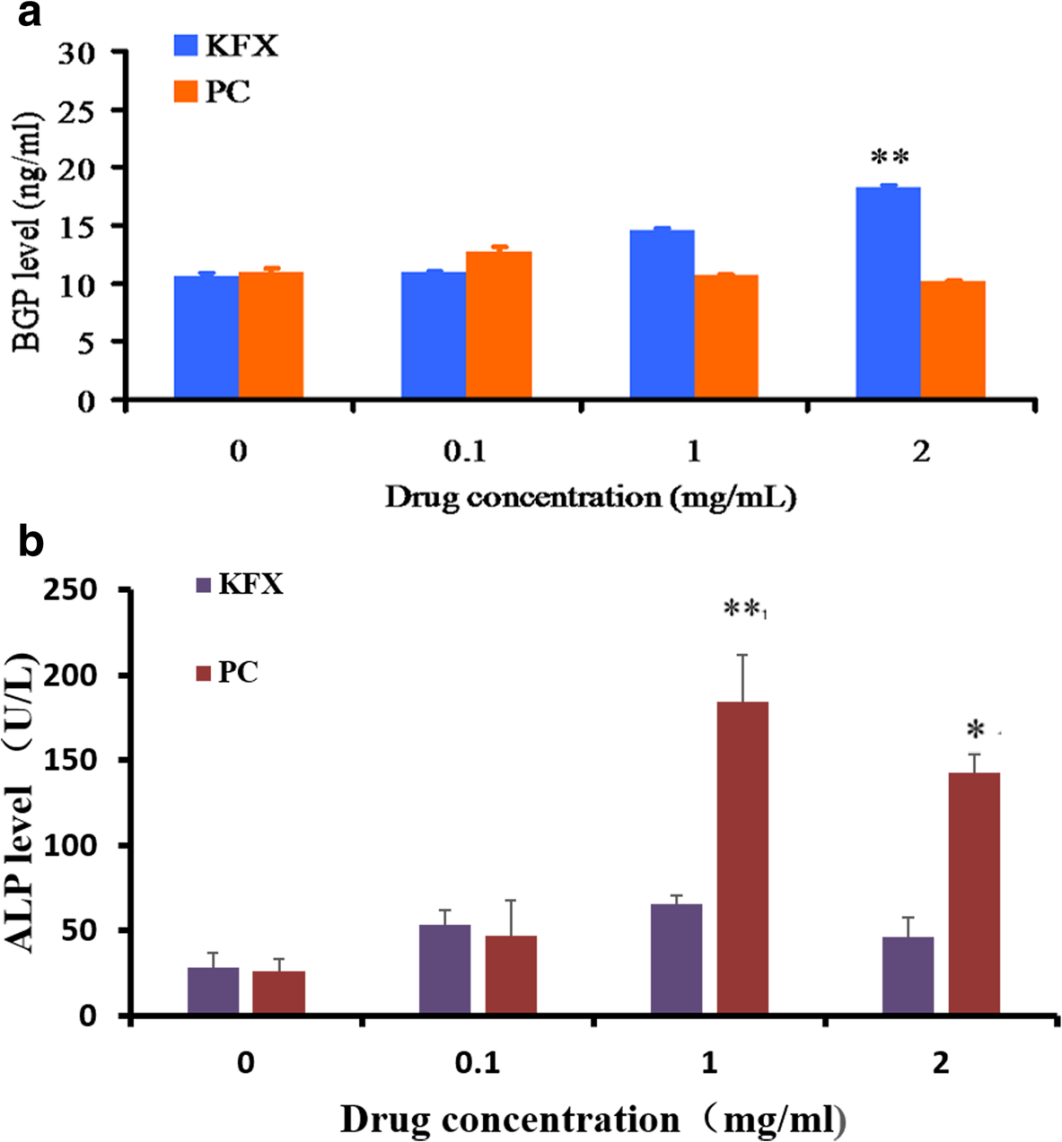
(**a**) Osteocalcin concentration, and (**b**) ALP activity in osteoblasts incubated with KFX and PC substance were determined. KFX treated group with the concentration of 2 mg/mL exhibit higher BGP level than PC substance. Data from three independent assays are presented as the mean ± SD (*n* = 3). Statistical significance was calculated by using t -test, * represents * *p* < 0.05,**p < 0.01

Osteocalcin is a factor in maintaining a normal bone calcification rate and inhibiting the cartilage mineralization rate, whose concentration can reflect the rate of bone formation and osteoblast activity. Increasing evidence has shown that BGP level is closely related to the bone formation. The extent of BGP level was quantified and presented as a histogram in Fig. 3a, it can be see that the PC substance (1, 2 mg/mL) group exhibit lower BGP level than the KFX treated group, with the significance identified at the 2 mg/mL of KFX treated group in contrast to the PC substance group (*p* < 0.01). The ALP activity of osteoblasts was examined. As shown in Fig. 3b, the quantitative analysis revealed that the ALP activity of the KFX treated group has not significantly increased. ALP activity of the cells cultured in PC substance was higher than that in KFX. This noticeable increased BGP level and no influence on ALP activity give further evidence for the significant stimulation of KFX in promoting calcification and inhibiting cartilage mineralization for the bone repair and regeneration.

### Effect of KFX on osteoclast apoptosis

**Fig. 4:**
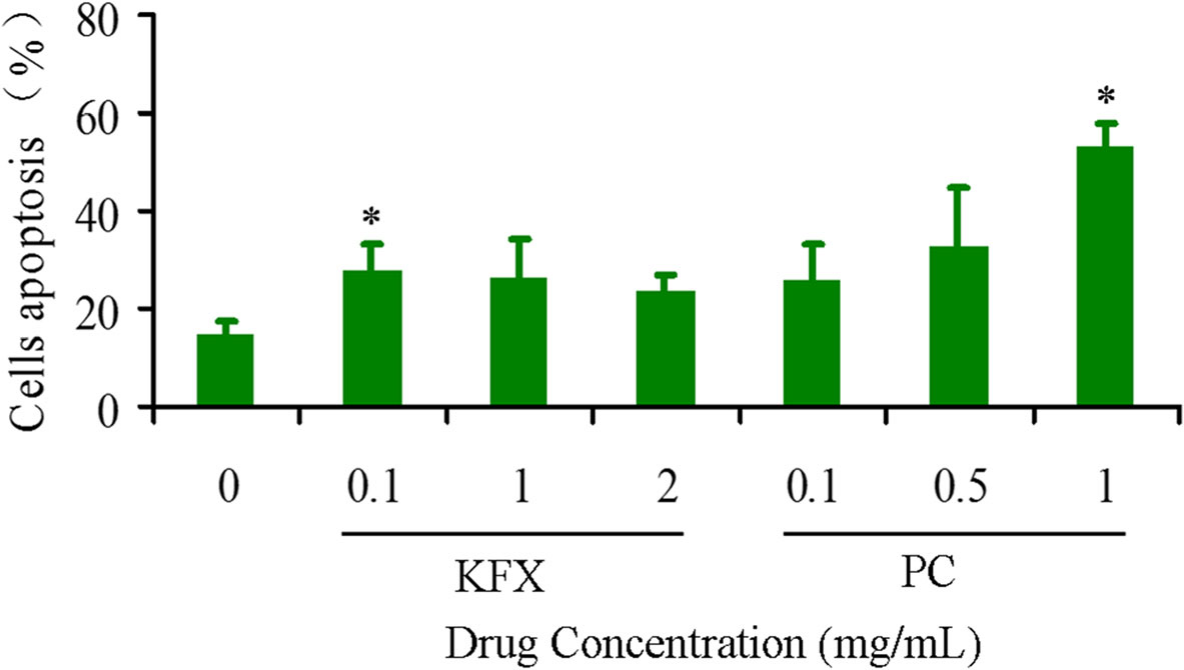
KFX and PC substance induce apoptosis in osteoclasts. Cells are incubated with various concentrations of KFX and PC substance for 72 h, and then the induction of apoptosis is determined by Annexin V-FITC/PI staining analysis. Cell apoptosis data are quantified and presented as a histogram. Data from three independent assays are presented as the mean ± SD (*n* = 3). * represents * *p* < 0.05,**p < 0.01

Osteoclasts play a pivotal role in bone resorption and are key factors in many bone disorders such as osteoporosis. Besides decreasing osteoclast formation, accelerating the death rate of osteoclasts is one of the widely used strategies to decrease bone resorption. We so tested if the influence of KFX in the osteoclasts apoptosis. As shown in Fig. 4, we observed the significant osteoclast apoptosis induced by KFX (0.1 mg/mL) or PC substance (1 mg/mL) treatment, compared with the blank control group. These results reminded that KFX can inhibit osteoclastic bone resorption and promote bone turnover, at least in part, by inducing osteoclast apoptosis.

### Effect of KFX on endothelial cells (HUVECs)

#### In vitro chemotaxis assay

**Fig. 5:**
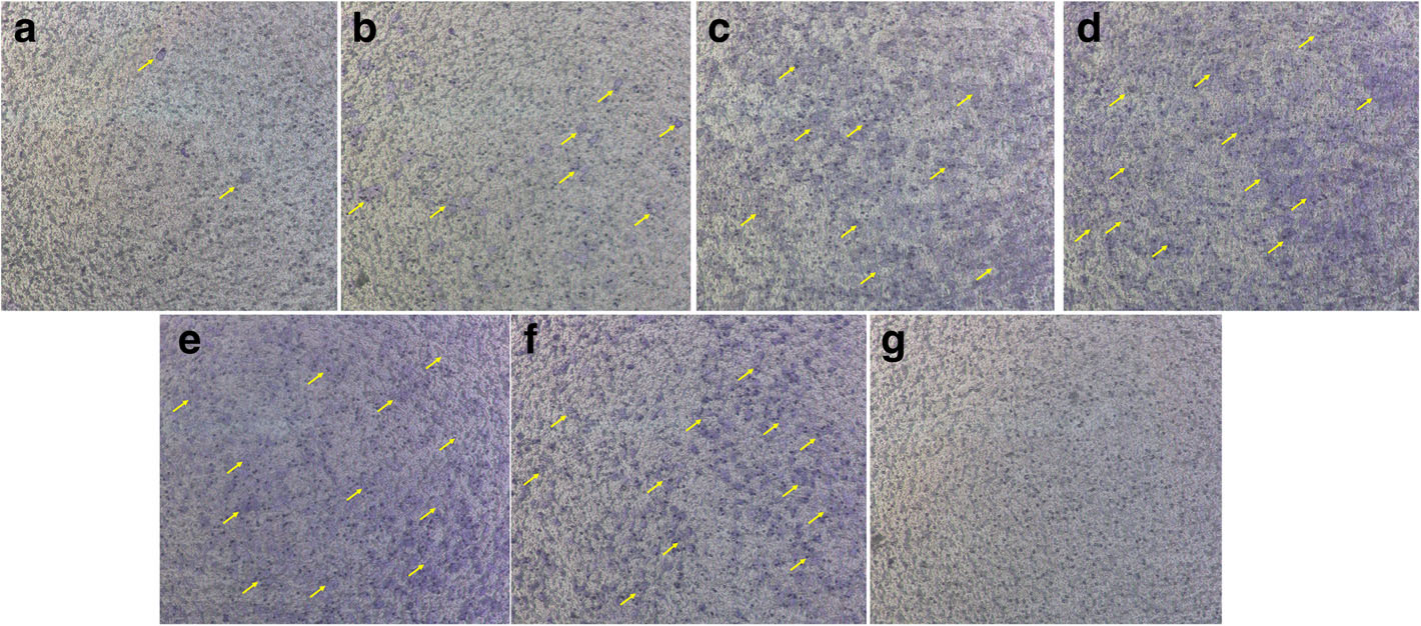
KFX and PC substance promote HUVECs migration. The migration properties of HUVECs are measured using a transwell migration assay. **a** Blank control (untreated), **b** KFX (0.1 mg/mL), **c** KFX (1 mg/mL), **d** KFX (5 mg/mL), **e** PC substance (0.1 mg/mL), **f** PC substance (1 mg/mL), **g** PC substance (5 mg/mL). Migrating endothelial cells are indicated by yellow arrows

Angiogenesis, the growth of new capillaries from preexisting blood vessels, is a complex process involving endothelial cell (EC) activation, disruption of vascular basement membranes, and migration and proliferation of ECs. It plays a crucial role in many biological processes including reproduction, development and repair. Migration is one of the preconditions of the angiogenesis process. In this study, the effect of KFX on endothelial cells migration was firstly examined by performing transwell assay. As shown in Fig. 5, endothelial cells treated with KFX (1, 5 mg/mL, Fig. 5b–d) and PC substance (0.1, 1 mg/mL, Fig. 5e, f) were displayed the significant higher migration rates in contrast to the blank control group (Fig. 5a).

#### Tube formation assay

**Fig. 6:**
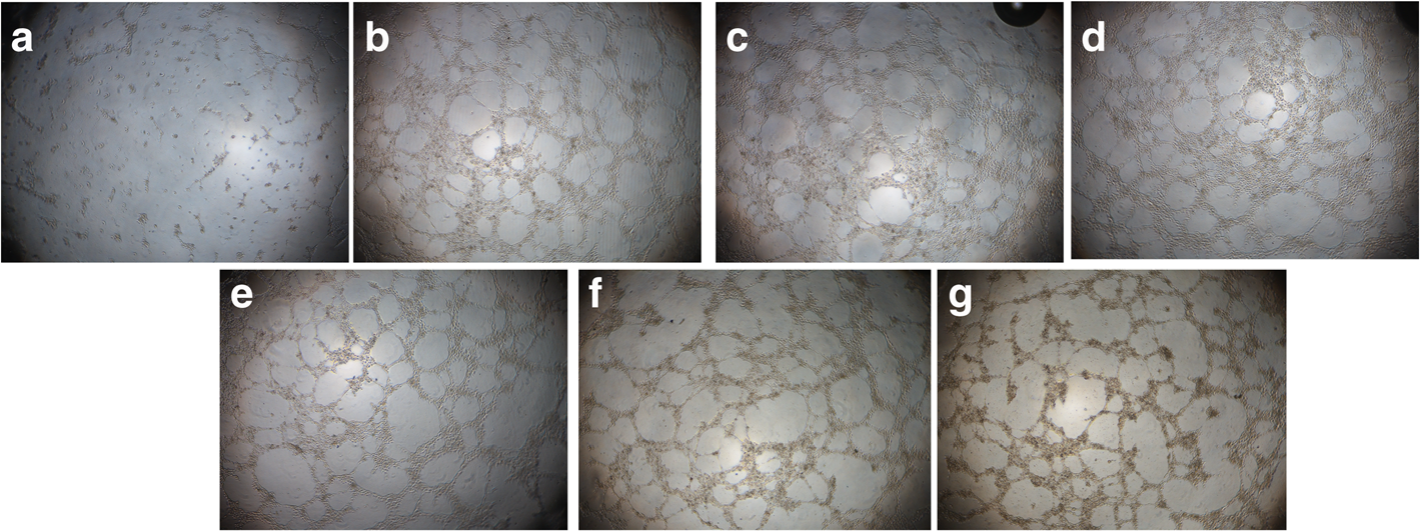
Tube formation assay in HUVECs incubated with KFX and PC substance. The morphological changes of the cells treated with different concentrations of KFX and PC substance are observed and photographed using a digital microscope. **a** Blank control (untreated), **b** KFX (0.1 mg/mL), **c** KFX (1 mg/mL), **d** KFX (2 mg/mL), **e**PC substance (0.1 mg/mL), **f** PC substance (1 mg/mL), **g** PC substance (2 mg/mL). Results indicate the endothelial cell tube formation in presence of KFX and PC substance suggesting their pro-angiogenic activities

HUVEC tube-formation assay is one of the simple, but well-established in vitro angiogenesis assays based on the ability of HUVECs to form three-dimensional capillary-like tubular structures, when cultured on a gel of growth factor-reduced basement membrane extracts. During the assay, HUVECs differentiate, directionally migrate to align, branch, and form the tubular polygonal networks of blood vessels. The tube-formation ability of HUVECs upon KFX treatment was investigated. As shown in Fig. 6, the tube formation assay in endothelial cells treated by KFX (Fig. 6b–d) and PC substance (Fig. 6e–g) have showed the obvious tubes, compared with the scarce and randomly distributed HUVECs of blank control, in which, no tube has been formed. These results together with the HUVEC migration assay results provided definite evidence that KFX has the strong effects in activating angiogenesis, which will be supply a combination effect for osteoporosis treatment.

### Effect of KFX on BMSCs

#### Cell proliferation and cell cycle analysis

**Fig. 7:**
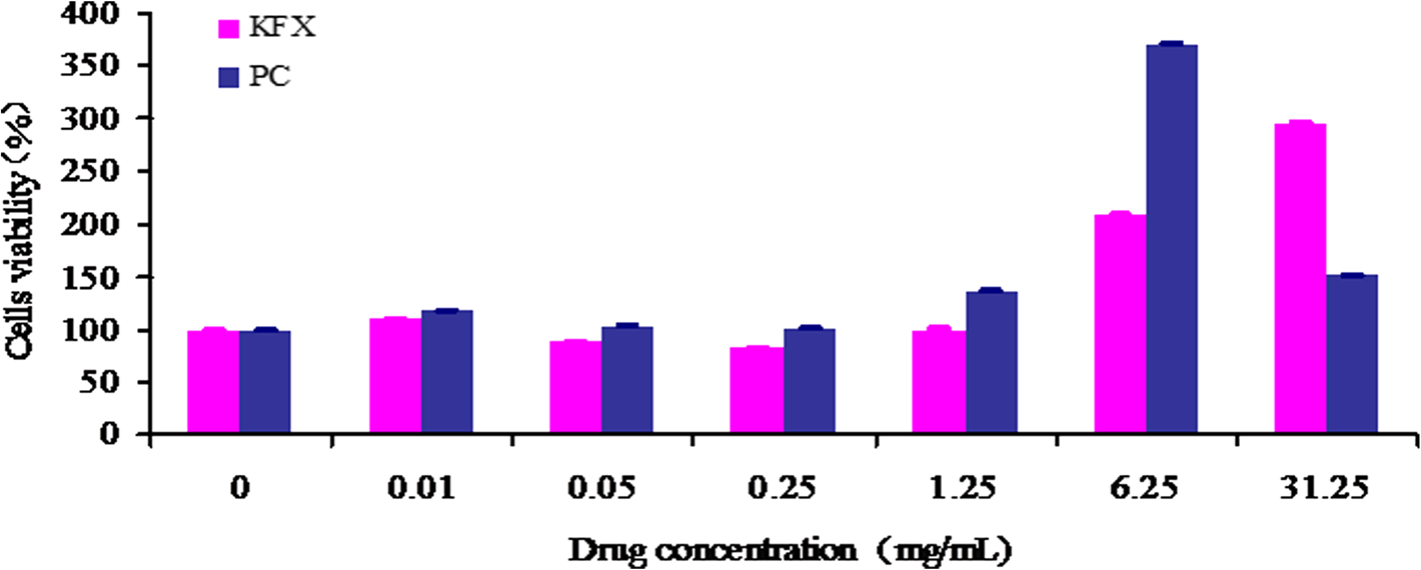
Cell viability assay (MTT assay) in BMSCs incubated with KFX and positive control in a dose-dependent manner for 24 h. KFX (1.25, 6.25, 31.25 mg/mL) and PC (1.25, 6.25, 31.25 mg/mL) drug have expressed an upward trend in BMSCs proliferation, compared with that of blank control group. Data are means ± SD of six independent experiments and statistical significance was calculated by using t -test (* represents * *p* < 0.05,**p < 0.01)

BMSCs are known to differentiate into various types of cells, including osteoblasts, fibroblasts, chondroblasts, and adipocytes [22], which has great roles in the bone recovery. In the present study, the influence of the KFX in the proliferation, cell cycle, as well as the migration of BMSCs was investigated. From the Fig. 7, upon the co-incubation of the BMSCs with KFX (0.01, 0.05, 0.25, 1.25, 6.25, 31.25 mg/mL) or positive control ((0.01, 0.05, 0.25, 1.25, 6.25, 31.25 mg/mL) for 24 h, both positive and KFX have expressed an upward trend in BMSCs proliferation, compared with that of blank control group. The significance was shown within 1.25–31.25 mg/mL by positive control, and at 31.25 mg/mL by KFX.

**Fig. 8:**
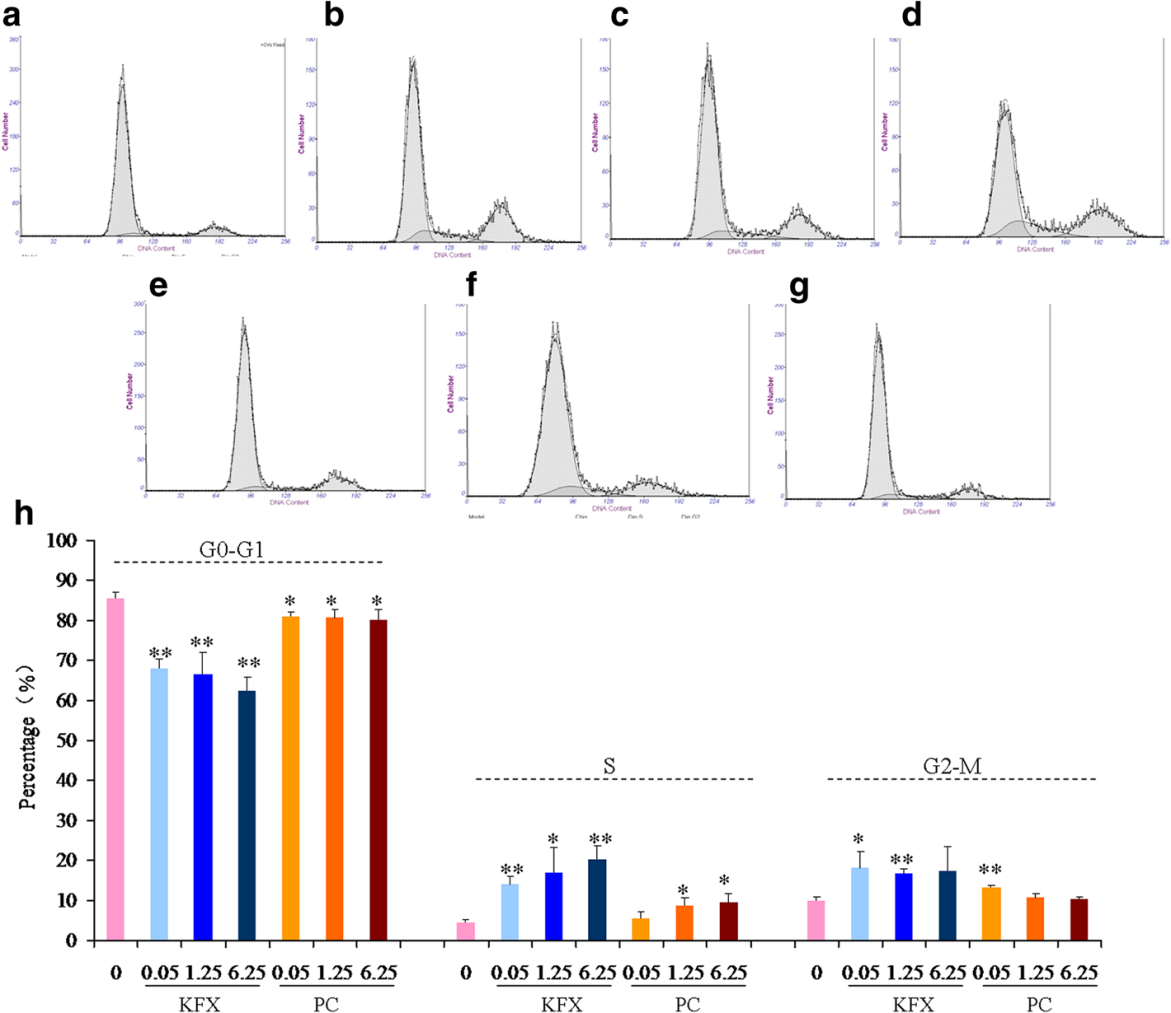
FACS or cell cycle analysis of BMSCs. **a** Blank control (untreated), **b** KFX (0.05 mg/mL), **c** KFX (1.25 mg/mL), **d** KFX (6.25 mg/mL), **e** PC substance (0.05 mg/mL), **f** PC substance (1.25 mg/mL), **g** PC substance (6.25 mg/mL), **h** Extent of propidium iodide staining of the gated population was displayed in a histogram and the regions are defined as: G0/G1, S, and G2/M. Result shows the effect of KFX and PC substance in BMSCs population of G0-G1, S and G2-M cell cycle phase, compared with that of blank control group. Data are means ± SD of three independent experiments and Statistical significance was calculated by using t –test (* *p* < 0.05,**p < 0.01)

Cell cycle is a progressive event for the division of mother cells to daughter cells through the pathway of a series of events consisting of G_0_-G_1_, S, G_2_/M phase [23]. Using fluorescence activated cell sorting (FACS), we further tested and observed a significantly fast cell cycle progression in KFX and positive control treated BMSCs than those of blank control (Fig. 8). The FACS result showed the extremely significant decrease of the cell population in G0-G1 phase in BMSCs upon the KFX (0.05, 1.25, 6.25 mg /mL) treatment (*p* < 0.01). Meanwhile, extremely significant increase in G2/M phase was expressed by the KFX treatment (0.05, 1.25, 1.25 mg/mL) (*p* < 0.01 or *p* < 0.05). Also, there is a significant increase in cell cycle population of S phase in BMSCs treated with KFX (0.05–6.25 mg /mL). Positive control treatment (0.05, 1.25, 6.25 mg/mL) also expressed the similar activities in decrease the cell population of G0-G1 phase, while increase the cell population of G2/M and S phases. However, in contrast to the KFX treatment, the efficacy of positive treatment is lower. These cell cycles regulation of KFX also suggested its therapeutic application in anti-osteoporosis with the BMSCs as a target.

#### Cell migration assay

**Fig. 9:**
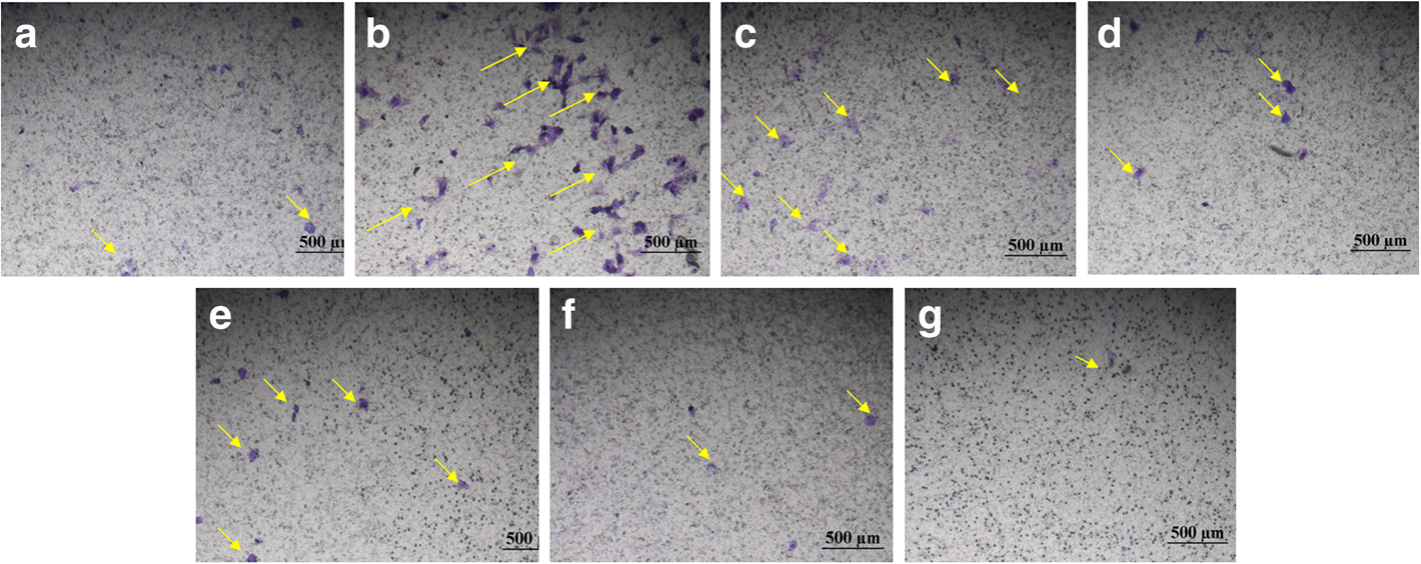
KFX and positive control promote BMSCs migration. The migration properties of BMSCs are measured using a transwell migration assay. **a** Blank control (untreated), **b** KFX (0.1 mg/mL), **c** KFX (1 mg/mL), **d** KFX (5 mg/mL), **e** PC substance (0.1 mg/mL), **f** PC substance (1 mg/mL), **g** PC substance (5 mg/mL). Migrating BMSCs are indicated by yellow arrows. Scale bar = 500 μm. Obvious migration was observed for BMSCs after treated with KFX (0.1 mg/mL)

The effect of KFX on BMSCs migration was further tested. As shown in Fig. 9, BMSCs treated with KFX (0.1, 1 mg/mL) were displayed a high migration rate compared with blank control group (Fig. 9a–c, migrating endothelial cells were indicated by yellow arrows). However, positive control treatment was not shown to give a significant influence in the BMSCs migration and few BMSCs have been identified for migration (Fig. 9e–g). The priority of KFX in the migration of BMSCs will facilitate the recovery of osteoporosis.

## Discussion

Post-menopausal osteoporosis is the most common bone disease, associated with low bone mineral density caused by an uncoupling of bone resorption from bone formation such that the activities of osteoclasts far outweigh those of the osteoblasts [24]. Osteoblasts could synthesize new collagenous organic matrix like BGP and regulate mineralization of matrix. During the bone formation period, the osteoblasts will express numerous markers such as BGP and various cytokines. BGP is a non-collagenous proteins present in bone tissue, which is the specific marker of bone formation and mostly used as indicators in evaluation of bone formation and bone turnover, and it can maintain normal bone mineralization rate and inhibit cartilage mineralization rate, thereby maintaining bone mass balance involved in bone remodeling [25, 26].

This study is first investigation to define the role of osteoblasts treated with KFX showed a significant migration to the bone pit and lay down the organic matrix to form solid bone, completing the bone remodeling process. And the positive control drug was the Xianlingubao capsule, which is an herbal formula based commercially available product and widely used in clinic for osteoprosis treatment [27]. Alizarin red S staining and BGP level assay are used as a measure of the ability of osteoblasts to mineralize the collagen matrix. The results above demonstrated that osteoblasts treated with KFX possessed significantly higher rate of mineralization compared with other tested groups. Hence, KFX were shown to be able to significantly stimulate bone formation by enhancing osteoblastic migration, increasing osteoblasts activity and inducing the formation of calcified nodules. Osteoclasts are multinucleated cells that branch from the monocyte or macrophage lineage. When osteoclastic bone resorption exceeds osteoblastic bone formation, bone density decreases, which can lead to osteoporosis. In this study, results showed that KFX is efficient in enhancing the apoptosis of osteoclasts and might be act as an promising agent for the treatment of osteoclasts induced bone resorptive disorders. To regenerate the osteoporotic defects and the bone defects with critical size, the angiogenesis and neovascularization are critical processes which provide nutrients and renewable autologous cells to heal the defect. In the present study suggested that KFX have also been shown to enhance the migration of HUVECs, and accelerated the angiogenesis process as compared with the blank control group. In addition, the migration assay of HUVECs demonstrated that KFX could induce the migration of HUVECs to bone defect site and then provide nutrients for renewable autologous cells.

Previous studies have shown that endogenous BMSCs played important roles in regulating the bone regeneration process, and it possesses the self-renewal capacity and the capability to differentiate into osteoblasts [14, 28, 29]. It is realized that the modulation of the in vivo differentiation of BMSCs toward osteoblasts and inhibition of osteoclastogenesis, accompanying with the promotion of the angiogenic activity of endothelial cells (ECs) are essential for osteoporotic bone regeneration [30–32]. In the present study, our results showed that the KFX is capable in stimulating the proliferation and migration of BMSCs comparing with the culture medium alone. Based on the findings in the current and previous research, it was concluded that KFX has the effect of promoting the migration of BMSCs to the osteoporotic site. At the same time, it also increases the number of BMSCs during the differentiation of BMSCs into osteoblasts. Therefore, the current findings will be helpful in future studies that aim to study the effectiveness of KFX in treating of osteoporosis.

## Conclusions

In this study, the in vitro anti-osteoporosis effects of KFX (*Periplaneta americana* L ethanol extract) in osteoblasts, osteoclast, HUVEC and BMSCs were comprehensively investigated. It was found that KFX has the significant 1) Acceleration of the migration of osteoblasts, HUVEC, and BMSCs; 2) Increase the secretion of osteocalcin and mineralization of osteoblasts; 3) Apoptosis of osteoclasts; 4) The proliferation and cell cycle regulation in BMSCs. These are the first reported evidence for the osteogenesis, anti-osteoporosis and angiogenesis effects of KFX, with the mechanism disclaimed to be related with activating the osteoblasts and HUVECs, as well as inhibit the activity of osteoclasts. The *Periplaneta americana* L extract was definitely shown to be a promising bone turnover agent with great potential for anti-osteoporosis treatment.

## Abbreviations

ARS: Alizarin red S
BGP: Bone gla protein
BMSCs: Bone marrow mesenchymal stem cells
DMSO: Dimethyl sulfoxide
EC: Endothelial cell
ECM: Extracellular matrix
FACS: Fluorescence activated cell sorting
GAP: Good Agriculture Practice
HUVEC: Human umbilical vein endothelial cell
KFX: Kangfuxin
MTT: 3-[4,5-dimethylthiazol-2-yl]-2,5-diphenyltetrazoliumbromide
PC: Positive control
SD: Standard deviation
TCM: Traditional Chinese Medicine
UPLC: Ultra Performance Liquid Chromatography

## References

[1] Kelchtermans H, Geboes L, Mitera T, Huskens D, Leclercq G, Matthys P (2009). Activated CD4+CD25+ regulatory T cells inhibit osteoclastogenesis and collagen-induced arthritis. Ann Rheum Dis.

[2] Campbell AJ, Buchner DM (1997). Unstable disability and the fluctuations of frailty. Age Ageing.

[3] Turner RT, Riggs BL, Spelsberg TC (1994). Skeletal effects of estrogen. Endocr Rev.

[4] Loder EW, Buse DC, Golub JR (2005). Headache as a side effect of combination estrogen-progestin oral contraceptives: a systematic review. Am J Obstet Gynecol.

[5] Ingle JN (2013). Postmenopausal women with hormone receptor-positive breast cancer: balancing benefit and toxicity from aromatase inhibitors. Breast.

[6] Abrahamsen B (2010). Bisphosphonate adverse effects, lessons from large databases. Curr Opin Rheumatol.

[7] Xia B, Xu B, Sun Y, Xiao L, Pan J, Jin H, Tong P (2014). The effects of Liuwei Dihuang on canonical Wnt/beta-catenin signaling pathway in osteoporosis. J Ethnopharmacol.

[8] Das S, Crockett JC (2013). Osteoporosis - a current view of pharmacological prevention and treatment. Drug Des Dev Ther.

[9] Carmeliet P, Jain RK (2011). Molecular mechanisms and clinical applications of angiogenesis. Nature.

[10] Li H, Chang J (2013). Stimulation of proangiogenesis by calcium silicate bioactive ceramic. Acta Biomater.

[11] Li H, Daculsi R, Grellier M, Bareille R, Bourget C, Remy M, Amedee J (2011). The role of vascular actors in two dimensional dialogue of human bone marrow stromal cell and endothelial cell for inducing self-assembled network. PLoS One.

[12] Terman BI, DougherVermazen M (1996). Biological properties of VEGF/VPF receptors. Cancer Metastasis Rev.

[13] Breier G, Clauss M, Risau W (1995). Coordinate expression of vascular endothelial growth factor receptor-1 (flt-1) and its ligand suggests a paracrine regulation of murine vascular development. Dev Dyn.

[14] Yang F, Yang D, Tu J, Zheng Q, Cai L, Wang L (2011). Strontium enhances osteogenic differentiation of mesenchymal stem cells and in vivo bone formation by activating Wnt/catenin signaling. Stem Cells.

[15] Fong EL, Chan CK, Goodman SB (2011). Stem cell homing in musculoskeletal injury. Biomaterials.

[16] Tang CH, Hsu TL, Lin WW, Lai MZ, Yang RS, Hsieh SL, Fu WM (2007). Attenuation of bone mass and increase of osteoclast formation in decoy receptor 3 transgenic mice. J Biol Chem.

[17] Song H, Wang W, Zhao P, Qi Z, Zhao S (2014). Cuprous oxide nanoparticles inhibit angiogenesis via down regulation of VEGFR2 expression. Nano.

[18] Peng LH, Huang YF, Zhang CZ, Niu J, Chen Y, Chu Y, Jiang ZH, Gao JQ, Mao ZW (2016). Integration of antimicrobial peptides with gold nanoparticles as unique non-viral vectors for gene delivery to mesenchymal stem cells with antibacterial activity. Biomaterials.

[19] Peng LH, Wei W, Qi XT, Shan YH, Zhang FJ, Chen X, Zhu QY, Yu L, Liang WQ, Gao JQ (2013). Epidermal stem cells manipulated by pDNA-VEGF165/CYD-PEI nanoparticles loaded gelatin/beta-TCP matrix as a therapeutic agent and gene delivery vehicle for wound healing. Mol Pharm.

[20] Korting HC, Schindler S, Hartinger A, Kerscher M, Angerpointner T, Maibach HI (1994). MTT-assay and neutral red release (NRR)-assay: relative role in the prediction of the irritancy potential of surfactants. Life Sci.

[21] Mukherjee S, Chowdhury D, Kotcherlakota R, Patra S (2014). B V, Bhadra MP, Sreedhar B, Patra CR. Potential theranostics application of bio-synthesized silver nanoparticles (4-in-1 system). Theranostics.

[22] Vanella L, Sanford C, Kim DH, Abraham NG, Ebraheim N (2012). Oxidative stress and heme oxygenase-1 regulated human mesenchymal stem cells differentiation. Int J Hypertens.

[23] Logan PC, Steiner M, Ponnampalam AP, Mitchell MD (2012). Cell cycle regulation of human endometrial stromal cells during decidualization. Reprod Sci.

[24] Clarke B (2008). Normal bone anatomy and physiology. Clin J Am Soc Nephrol.

[25] Verit FF, Geyikli I, Yazgan P, Celik A (2006). Correlations of serum prolidase activity between bone turnover markers and mineral density in postmenopausal osteoporosis. Arch Gynecol Obstet.

[26] Nian H, Ma MH, Nian SS, Xu LL (2009). Antiosteoporotic activity of icariin in ovariectomized rats. Phytomedicine.

[27] Lou H, Huang D, Fang S, Zhao X, Huang Q, Wang M (2009). Clinic research on Xianling Gubao capsules for treatment of manopause syndrome. Chin J Chin Materia Medica.

[28] Glynn ERA, Londono AS, Zinn SA, Hoagland TA, Govoni KE (2014). Culture conditions for equine bone marrow mesenchymal stem cells and expression of key transcription factors during their differentiation into osteoblasts. J Anim Sci Biotechnol.

[29] Abdallah BM, Jafari A, Zaher W, Qiu W, Kassem M (2015). Skeletal (stromal) stem cells: an update on intracellular signaling pathways controlling osteoblastdifferentiation. Bone..

[30] Peng SL, Liu XS, Wang T, Li ZY, Zhou GQ, Luk KDK, Guo XE, Lu WW (2010). In vivo anabolic effect of strontium on trabecular bone was associated with increased osteoblastogenesis of bone marrow stromal cells. J Orthop Res.

[31] Neve A, Cantatore FP, Corrado A, Gaudio A, Ruggieri S, Ribatti D (2013). In vitro and in vivo angiogenic activity of osteoarthritic and osteoporotic osteoblasts is modulated by VEGF and vitamin D3 treatment. Regul Pept.

[32] Guan M, Yao W, Liu RW, Lam KS, Nolta J, Jia JJ, Panganiban B, Meng LP, Zhou P, Shahnazari M (2012). Directing mesenchymal stem cells to bone to augment bone formation and increase bone mass. Nat Med.

